# A Deep Learning Framework for Accurate Vehicle Yaw Angle Estimation from a Monocular Camera Based on Part Arrangement

**DOI:** 10.3390/s22208027

**Published:** 2022-10-20

**Authors:** Wenjun Huang, Wenbo Li, Luqi Tang, Xiaoming Zhu, Bin Zou

**Affiliations:** 1 Foshan Xianhu Laboratory of the Advanced Energy Science and Technology Guangdong Laboratory, Xianhu Hydrogen Valley, Foshan 528200, China; 2 Hubei Key Laboratory of Advanced Technology for Automotive Components, Wuhan University of Technology, Wuhan 430070, China; 3 Hubei Collaborative Innovation Center for Automotive Components Technology, Wuhan University of Technology, Wuhan 430070, China; 4 Hubei Research Center for New Energy and Intelligent Connected Vehicle, Wuhan 430070, China

**Keywords:** pose estimation, yaw angle estimation, convolutional neural network, part arrangement, monocular camera

## Abstract

An accurate object pose is essential to assess its state and predict its movements. In recent years, scholars have often predicted object poses by matching an image with a virtual 3D model or by regressing the six-degree-of-freedom pose of the target directly from the pixel data via deep learning methods. However, these approaches may ignore a fact that was proposed in the early days of computer vision research, i.e., that object parts are strongly represented in the object pose. In this study, we propose a novel and lightweight deep learning framework, YAEN (yaw angle estimation network), for accurate object yaw angle prediction from a monocular camera based on the arrangement of parts. YAEN uses an encoding–decoding structure for vehicle yaw angle prediction. The vehicle part arrangement information is extracted by the part-encoding network, and the yaw angle is extracted from vehicle part arrangement information by the yaw angle decoding network. Because vehicle part information is refined by the encoder, the decoding network structure is lightweight; the YAEN model has low hardware requirements and can reach a detection speed of 97FPS on a 2070s graphics cards. To improve the performance of our model, we used asymmetric convolution and SSE (sum of squared errors) loss functions of adding the sign. To verify the effectiveness of this model, we constructed an accurate yaw angle dataset under real-world conditions with two vehicles equipped with high-precision positioning devices. Experimental results prove that our method can achieve satisfactory prediction performance in scenarios in which vehicles do not obscure each other, with an average prediction error of less than 3.1° and an accuracy of 96.45% for prediction errors of less than 10° in real driving scenarios.

## 1. Introduction

Pose estimation is an important topic in computer vision and a key technology in fields such as autonomous vehicles [1] and video surveillance [2]. Image algorithm researchers pursue information about the shape [3], distance [4,5], velocity [6,7], position, and orientation [8,9] of objects. In terms of pose estimation, objects are often classified as humans [10,11,12] and rigid bodies depending on whether they are deformable or not. The pose estimation task for rigid bodies can be traced back to the very early stages of computer vision [13], comprising six degrees of freedom (DoFs; X,Y,Z,α,β,γ). Rigid body pose estimation has a significant component of the field of unmanned operations, e.g., industrial robots and autonomous vehicles. In the case of autonomous vehicles, for example, accurate vehicle posture estimation is important to the achievement of self-driving, as the future trajectory of objects can be predicted [14], and their states can be analyzed based on the current pose of the vehicle. Thus, a framework that can accurately estimate the poses of vehicles is critically needed.

Studies on object acquisition from monocular RGB images can be categorized according to whether they use a data-driven methodologies. The main idea of the non-data-driven approach is to match the object image taken by the camera with template images (this template can be either CAD images [15] or real images [16]) to obtain a prediction result, and the matching elements can comprise classical image features, such as SIFT, SURF [17], etc. Methods using data-driven approaches can be divided into those based on detection of key points and those based on end-to-end learning. The former constructs a 2D–3D correspondence by matching the image with a virtual 3D model and then solving the target pose via the perspective-n-point (PnP) [18] method, whereas the latter regresses the six-degrees-of-freedom (6DoF) poses of the target directly from the pixel data by extracting image features through convolutional neural networks. These methods may ignore a fact that was proposed in the early days of computer vision research, i.e., that the object parts are strongly represented in the object pose. Taking the surrounding vehicles as the observation target, a mapping relationship can be obtained between the poses of the vehicle and the arrangement of the part positions based on image observation. For example, when a vehicle in the image has an attitude in the same direction as ours, we will observe two taillights. Similarly, when we observe two taillights, we can roughly estimate that the vehicle is oriented in the same direction as us. Here, we proposed a novel framework for prediction of yaw angle using deep neural networks to learn the mapping relationship between part position arrangements and the object pose.

**Figure 1:**
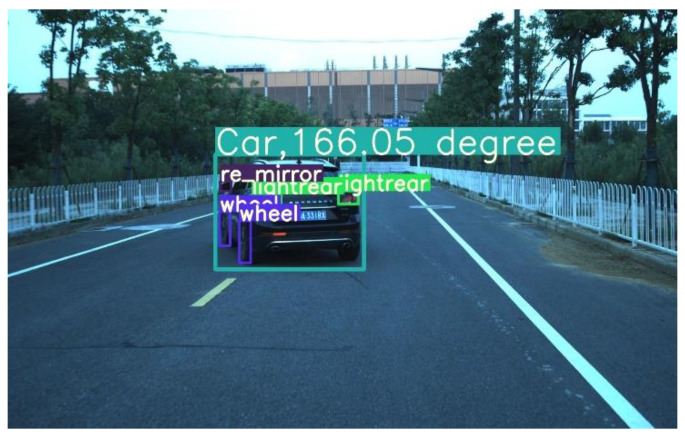
Illustration of YAEN-predicted yaw angle on a real road.

The proposed framework based on part arrangement for accurate yaw angle estimation is called YAEN (yaw angle estimation net). YAEN views vehicles as objects and their wheels, front lights, taillights, and rearview mirrors as parts (Figure 1). We selected the vehicle yaw angle as the research object because, on the one hand, it has a greater impact on the vehicle trajectory than the roll angle and the pitch angle of the vehicle [19,20]. On the other hand, the currently widely used yaw angle detection methods mainly rely on LIDAR cluster analysis of. However, the LIDAR detection effect is not sufficient in rain and snow conditions, in addition to too expensive [21] for use in daily life. Therefore, the existing methods cannot be widely applied in the short term.

The proposed framework for yaw angle prediction consists of a part-encoding network and a yaw angle decoding network. In the former, the object and the parts are detected from an image by an advanced object detector [22,23], then encoded. We consider each part as a “material” to express the object pose, and the arrangement of “materials” can convey semantic information about the object poses. In the latter, the “materials” information is extracted by a deep neural network decoder to obtain the pose information of the object. To verify the effectiveness of YAEN, we constructed an accurate real-world yaw angle dataset involving two vehicles equipped with high-precision positioning equipment. Experimental results show that YAEN can quickly and accurately detect the surrounding vehicles and predict vehicle yaw angles using a monocular camera.

The remainder of this paper is organized as follows. In Section 2, we review work by other scholars on pose estimation and vehicle pose datasets. The collection and processing methods of the yaw angle dataset are introduced in Section 3. In Section 4, we discuss the structure and loss function design of the YAEN network. We present the experimental results of YAEN on the dataset in Section 5. Finally, in Section 6, we present our study conclusions.

## 2. Related Work

The yaw angle estimation problem is a subproblem of pose estimation. In this section, we review recent studies on pose estimation and relevant datasets and describe the relationship between these studies and our work.

### 2.1. Pose Estimation

In recent years, an increasing number of studies has been published on pose estimation. Researchers have used various sensors to obtain sufficient information to estimate object poses.

For pose estimation tasks, LIDAR is advantageous because the acquired point cloud data contain distance information. With such point cloud data, several authors have used clustering and template-matching methods to predict object poses [24,25,26,27,28]. However, owing to its high cost, LIDAR cannot be widely applied in the short term, so some researchers have used cameras to estimate object poses.

The methods of estimating object pose from images can be broadly classified into two forms. The first form involves the construction of a 2D–3D correspondence by matching images with 3D model renderings and then using the perspective-n-point method [18] to solve the object pose [9,29,30]. The second form involves obtaining the target object’s six-degrees-of-freedom (6-DoF) pose directly from the pixel data [8,10,31,32,33]. The disadvantage of the first form is that the construction of a 2D–3D correspondence is susceptible to feature changes, so depth cameras are often introduced as auxiliary information [34]. This problem can be circumvented by the second form, which obtains the object pose directly from image pixels without requiring the construction of a correspondence between the images and the 3D models. In the method of obtaining the object pose directly from the image, the feature information is first extracted from the image and decoded to extract the rotation and translation information about the object, ultimately obtaining the object pose. The feature information can comprise either the 2D BBox acquired by the object detector [22,23,35,36] or heatmaps of the object obtained by the key point detection network [33,37,38].

For example, the SSD-6D network proposed by Kehl [8] is an extension of the object detection model SSD [23]. In addition to the object 2D BBox, more specific 2D BBoxes can be predicted by adding an inception module [39]; these 2D BBoxes can be combined to obtain the 3D BBox of the object. Wu et al. [40] obtained a vehicle heatmap by Mask RCNN and added a head structure to predict the vehicle rotation and translation vectors.

The above attitude prediction algorithms can predict the 6-DoF information, but these predictions are only approximate. Even the 6D-VNet model [40], which obtained the best result in the Apolloscape challenge 3D Vehicle Instance task [41], is not sufficiently accurate. Moreover, the probability that the error of vehicle distance prediction is less than 2.8 m is only 35.3%, and the error of angle prediction is below 50°. Such results cannot be used to guide vehicles for effective autonomous driving.

### 2.2. Dataset of Vehicle Poses

To obtain an accurate pose estimation model, the dataset used for model training needs to be accurately annotated. Unfortunately, accurate annotation is costly and inaccessible. Currently available public datasets for vehicle pose estimation include KITTI Object 3D [42,43], ApolloVehicle3D [41,44], and PASCAL [45], all of which are produced without sensors installed on the observed vehicles.

To determine the pose of the observed vehicle, KITTI Object 3D creates an annotation tool [43] that displays both a 3D point cloud and an image to assist in manual annotation. In comparison, ApolloVehicle3D and PASCAL create a high-quality 3D model of the observed vehicle, with the key points of the vehicle manually annotated in the image; then, use EPnP algorithm [18] is used to obtain the 3D pose of the vehicle based on the 2D image.

Benefiting from these semi-automatic annotation method, a wealth of data can be obtained. However, the fact that the ground truths of KITTI Object 3D and ApolloVehicle3D for vehicle pose are indirectly inferred results in deficient accuracy. Therefore, in the present study, we constructed a new dataset, the Yaw Angle Dataset, which was acquired using two vehicles equipped with high-precision positioning equipment. This dataset can obtain the pose information of both vehicles directly through sensors without going complicated intermediate steps.

The conclusions drawn from the abovementioned related studies are summarized below. The goal of vehicle pose estimation research is to use a network to achieve 6-DoF estimation; however, it may be difficult to achieve accurate detection using six degrees of freedom. To solve the problem of coarse pose estimation, we propose a framework for accurate yaw angle estimation based on the arrangement of parts. The proposed yaw angle estimation network (YAEN) achieved an average prediction error or less than 3.1° and an accuracy of 96.45% for prediction errors of less than 10° in real driving scenarios.

In summary, the contribution of our work comprises three main areas.

- We propose a framework for accurate yaw angle estimation, YAEN, based on the arrangement of parts. YAEN can quickly and accurately predict the yaw angle of a vehicle based on a single RGB image;
- A novel loss function is proposed to deal with the problem caused by the periodicity of the angle; and
- To test the accuracy of our network, we created a vehicle yaw angle dataset—the Yaw Angle Dataset, which comprised 17,258 images containing 15,863 yaw angle annotations, 17,258 2D BBox annotations of vehicles, and 73,191 2D BBox annotations of parts of vehicle parts. This dataset was used to validate the effectiveness of YAEN.

## 3. Yaw Angle Dataset

To collect sufficient yaw angle data, the Yaw Angle Dataset was created using two vehicles equipped with high-precision positioning equipment. Two vehicles were used for all data collection. We collected many images and yaw angle data for various types of vehicles (sedans, SUVs, etc.) in daily traffic. In addition to collecting vehicle attitude information on regular roads, we collected a large amount of data that are difficult to collect on regular roads in a closed practice range environment. We also collected data under a wide range of road conditions, including daytime, evening, and rainy days.

### 3.1. Devices

**Figure 2:**
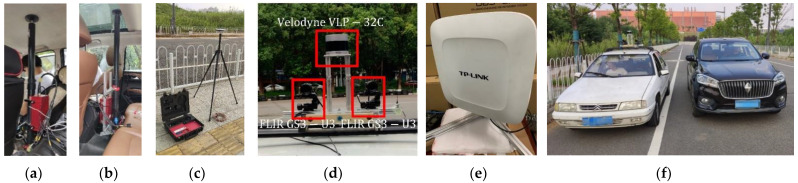
Various devices used for data collection, from left to right: (**a**) OXTSGPS RT3000 v2 mounted on the observation vehicle, (**b**) OXTSGPS RT3000 v2 mounted on the observed vehicle, (**c**) OXTS RT-BASE, (**d**) Velodyne VLP-32C and two FLIR GS3-U3 cameras, (**e**) TL- AP450GP, and (**f**) two vehicles.

The devices used to collect accurate data are shown in Figure 2. The role and related parameters of each device are shown in Table 1.

### 3.2. Data Collection

#### 3.2.1. Time Synchronization

The data collected here need to correspond precisely in series, which requires time synchronization. Depending on the object, time synchronization can be divided into time synchronization between different sensors of a single vehicle and time synchronization between sensors on different vehicles. To realize the former, we designed a trigger mechanism. All sensors were turned on at all times during data collection, and each sensor constantly refreshed the captured data. However, each sensor refreshed data at a specific frequency (e.g., 100 HZ for GPS, 160 HZ for cameras), so we designed a signal generator that runs continuously on a computer. The generator sends out a collection signal at a fixed frequency (e.g., 10 HZ), which contains the timestamp of the current moment and the collection command. Whenever a sensor receives a collection signal, it saves the data it is currently obtaining. For the latter time synchronization, all devices were connect to one LAN, with socket communication technology used to facilitate information acquisition by the devices.

#### 3.2.2. Collected Scenes

Through the above method, data can be collected from several driving scenarios on an open road, for example following, overtaking, and meeting driving scenarios. To perform these maneuvers, two drivers drive the observing vehicle and the observed vehicle, respectively, at the same time at a speed of less than 30 km/h to ensure the safety of the experiment. However, during normal driving, the vehicle’s yaw angle is not sufficiently variable, and the data are concentrated at some angle scales. In order for our model to recognize various yaw angles, the range of the collected yaw angle data must be 0° to 360°. To this end, we intentionally collected some data that do not occur on ordinary roads in a closed driving field. For example, the observed completed make a circle motion or a figure-eight loop in the driving field while the observing vehicle remained fixed in order form various yaw angles.

#### 3.2.3. Data Processing

**Figure 3:**
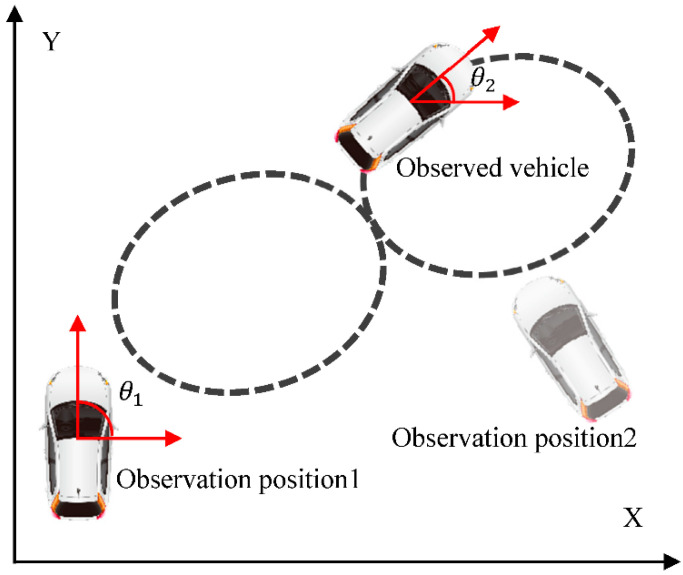
Illustration of observing yaw angle.

**Figure 4:**
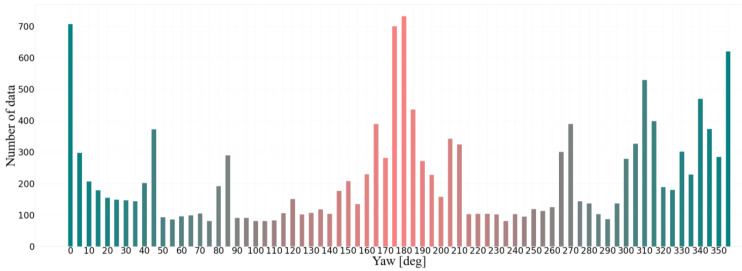
The distribution of yaw angles in the Yaw Angles Dataset, where each bar spans 5°. For instance, the leftmost and rightmost bars indicate the amount of data in the ranges of 0°–5°and 355°–360°, respectively. Bars of similar colors indicate that their yaw angles are close to real-world conditions.

The GPS we employed for data collection contains an IMU device, which can directly acquire the vehicle’s yaw angle with a yaw angle measurement error of 0.03°. However, the data collected through two GPSs are the yaw angles of the two vehicles in the geodetic coordinate system (GCS) (Figure 3, $0<\theta_{1},\;\theta_{2}\leq 360{}^{\circ}$). The yaw angle of surrounding vehicles can be obtained from the camera. Even if the position and pose of the vehicle do not change, the results may differ considerably depending on the observation angle. Therefore, the yaw angle of the observed vehicle in the GCS is converted to the yaw angle relative to the coordinates of the observing vehicle and expressed as θ. Thus, once θ is obtained, it can be simply converted to the yaw angle in the GCS. Equation (1) is used to express the angle conveniently to record θ :

(1) $$\theta =\{\begin{array}{ll} \theta_{1}-\theta_{2}+540, & \theta_{1}-\theta_{2}\leq -180\\ \theta_{1}-\theta_{2}+180, & \theta_{1}-\theta_{2}>-180\;\mathrm{and}\;\theta_{1}-\theta_{2}<180\\ \theta_{1}-\theta_{2}-180, & \theta_{1}-\theta_{2}\geq +180 \end{array}$$

where $\theta_{1}\;\mathrm{and}\;\theta_{2}$ denote the yaw angles of the observation and observed vehicles, respectively, in the GCS obtained by high-precision positioning equipment. As shown in Equation (1), the relative yaw angle between the two vehicles (θ) is a primary function of $\theta_{1}$ and $\theta_{2}$, so the measurement error of θ is 0.06°. Through the conversion of Equation (1), θ can be maintained between 0° and 360°, where θ=0° or 360° indicates that the observed vehicle is in oriented in the opposite direction to that of the observing vehicle, and when θ=180°, the two vehicles are oriented in the same direction. These two cases are the most common situations encountered under actual driving conditions (following and meeting), so our dataset is similar to ApolloCar3D [41] and KITTI Object 3D [42] datasets, with the highest percentage of data corresponding to following and meeting scenarios (Figure 4).

#### 3.2.4. Annotations

**Figure 5:**
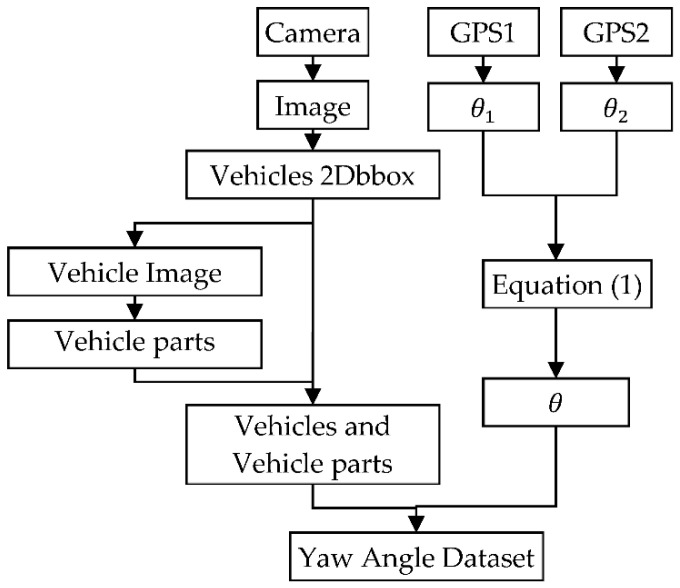
Flow chart of dataset annotation.

In addition to annotation of the vehicle yaw angle in each image, we also marked the positions of vehicles and vehicle parts in the image. We selected the following parts of the vehicle with apparent features to represent the vehicle posture: wheels, headlights, taillights, and rearview mirrors. The annotation of vehicles is relatively simple because the ratio of pixels occupied by vehicles is satisfactory; annotation can be performed using the labelImg labeling tool. However, the vehicle parts are small and thus cannot be easily labeled directly on the image, so we adopted a hierarchical labeling method (Figure 5). First, the vehicle is annotated on the complete image. Then, with the annotated result, we the image containing only the pixel portion of the vehicle is cut out, and the parts are annotated on the vehicle image. Finally, the parts annotated on the vehicle image are mapped onto the original image to determine the positions of the parts in the whole image. Because time synchronization was performed between the pictures taken by the camera and the yaw angle collected by the GPS, we combined the part positions and the relative yaw angle (θ) obtained from the two GPSs to obtain the Yaw Angle Dataset.

## 4. YAEN

**Figure 6:**
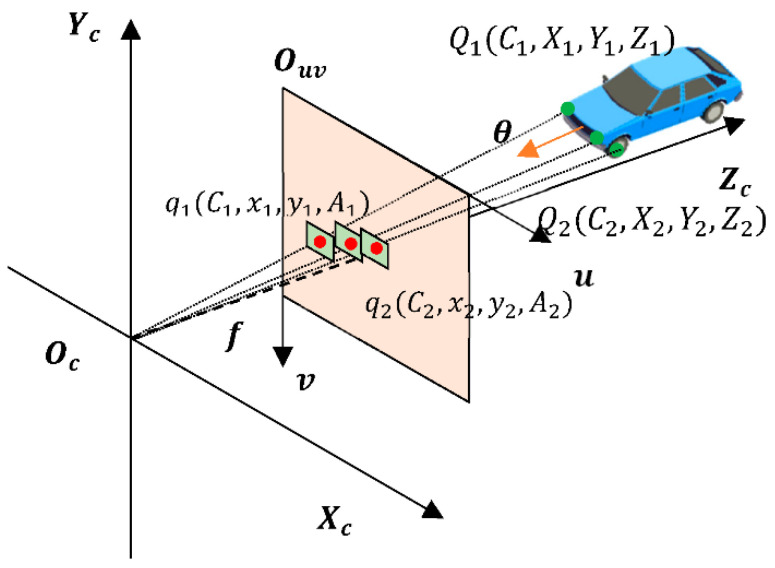
Illustration of the relationship between the vehicle yaw angle (*θ*) and the vehicle in a two-dimensional pixel coordinate system.

The proposed framework for accurate yaw angle estimation based on the arrangement of parts(YAEN) aims to derive the yaw angle of a vehicle (θ) in the world coordinate system from the image pixel. The image acquired by the camera is a projection of the three-dimensional world into two dimensions (Figure 6). Ignoring the deformation of the vehicle tires and suspension, we assume that the vehicle is a rigid body, and when the coordinates of three points of the vehicle that are not in the same line under the world coordinate system are fixed, the attitude of the vehicle under the world coordinate system is fixed. The yaw angle of the vehicle (θ) can be inferred from the inherent frame of the vehicle. Let this relationship be f.

(2) $$\theta =f(\{Q_{i}(C_{i},X_{i},Y_{i},Z_{i}),i=1,2\ldots \})$$

where θ denotes the yaw angle of the vehicle; $Q_{i}$ denotes point i in the 3D coordinate system, $C_{i}$ is the category of point i; and $X_{i},\;Y_{i},\;\mathrm{and}\;Z_{i}$ are the 3D coordinates of point i. The coordinates of the vehicle in the 3D coordinate system and the coordinates in the 2D pixel coordinate system can be connected by the internal camera parameters and the external camera parameters; this relationship is denoted as g.

(3) $$Q_{i}=g(q_{i}(C_{i},x_{i},y_{i},A_{i}))$$

where $Q_{i}$ denotes point i in the 3D coordinate system; $q_{i}$ denotes point i in the 2D pixel coordinate system; $C_{i}$ denotes the category of point i; $x_{i}\;\mathrm{and}\;y_{i}$ are the 2D coordinates of point i; and $A_{i}$ represents the pixel area occupied by the part to which point i belongs. By substituting Equation (3) into Equation (2), the relationship between the yaw angle (θ) and the 2D pixel coordinate points can be obtained, as shown in Equation (4).

(4) $$\theta =f(\{g(q_{i}(C_{i},x_{i},y_{i},A_{i})),i=1,2\ldots \})$$

The mapping relationship represented by Equation (4) is what we want YAEN to learn.

**Figure 7:**
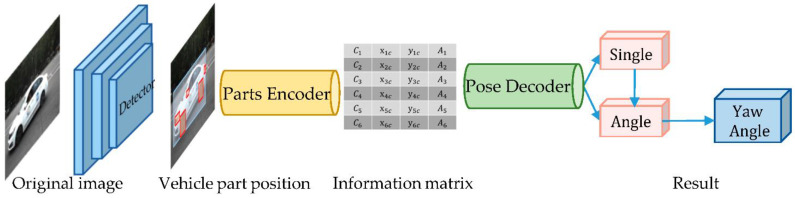
Illustration of the YAEN yaw angle estimation process. YAEN contains an object detector, a part encoder, and a part decoder.

We designed YAEN as an encoding–decoding structure consisting of two parts: a part-encoding network and a yaw angle decoding network. The former is used to encode the information (e.g., the position and size of the object parts) and to obtain the information matrix, which consists of an advanced object detector and a part encoder. The latter is used to decode the information matrix and calculate the object yaw angle. The estimation process of YAEN is shown in Figure 7.

Specifically, the input of YAEN is an RGB image. In the first step, this image is normalized to a fixed size (in this paper, the normalized image size is 640×640). In the second step, the normalized image is fed into a convolutional neural network for vehicle and vehicle part detection. In the third step, the detection results of the components are encoded. The coordinates, type, and size of the centroids of multiple parts are represented in an information matrix. Finally, the information matrix is input into the yaw angle decoding network to obtain the prediction results of the vehicle yaw angle. The model code is available at https://github.com/Hurri-cane/Yaw-angle-estimation-network (accessed on 8 September 2022).

### 4.1. Part-Encoding Network

The part-encoding network encodes the original image into the semantic “material” that constitutes the yaw angle. It consists of an advanced object detector and an encoder. Any type of advanced object detector [22,35,36] can be applied as needed. Assuming a preference for rapid encoding, we used a single-stage network as the object detector: YOLOv5-s [46]. This network can achieve high performance for vehicle and vehicle part detection (Table 2), with an *mAP* of up to 0.996, meeting the criteria set for the present study.

**Figure 8:**
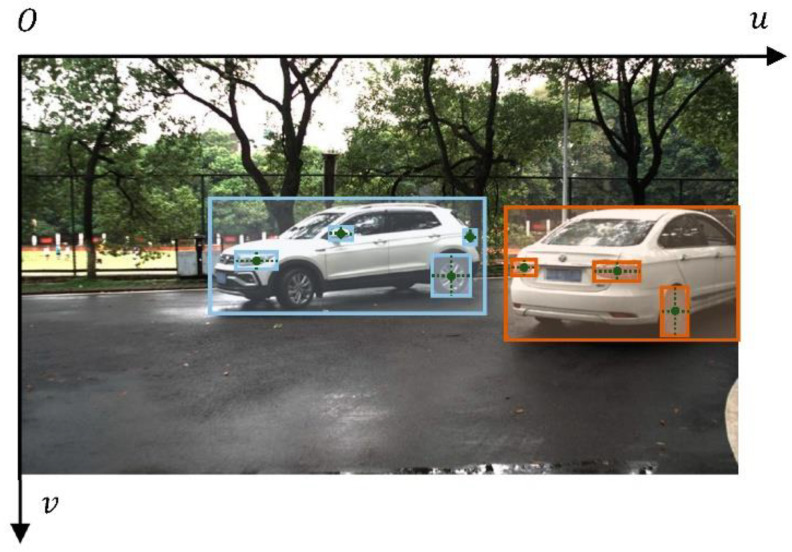
Illustration of part division process after the object detector detects vehicles and parts.

The object detector detects both vehicles and vehicle parts. When many vehicles are present in an image, the parts belonging to different vehicles must be categorized into different vehicles. We employed a bottom-up approach to construct different sets of vehicle parts. First, the 2D BBoxes of all vehicles and vehicle parts are extracted from the image. Then, the center positions of the parts are calculated based on the 2D BBoxes. If a center position falls into the 2D BBox of a given vehicle, then this part is classified into the corresponding vehicle. In this way, the 2D BBox of each vehicle part in the original image is obtained (Figure 8). This method does not perfectly solve the problem of part categorization, especially in cases in which vehicle obscuring each other; this method was selected as a compromise to deal with the multivehicle problem.

The encoder then encodes the vehicle parts obtain the information matrix. At this stage, the pixel information of the parts is discarded, and the category, position, and size information detected by the part detector are used directly. This allows the yaw angle decoder to obtain enough clear information to simplify the design of the yaw angle decoder. Experiments showed that this allows the yaw angle decoding network to achieve accurate decoding of the yaw angle with minimal computation. Taking part i of the vehicle as an example, the information of part i can be encoded as $\lambda_{i}=\left[C_{i},\;x_{ic},y_{ic},A_{i}\right]$, where$\;C_{i}$ is the category of part *I*, $x_{ic}\;\mathrm{and}\;y_{ic}$ are the center coordinates of part *I*, and $A_{i}$ is the relative size of the 2D BBox pixel area between parts. The encoding results of each part are concatenated vertically to form an information matrix (*M*). *M* contains the pose information of the vehicle:

$$M=\left(\begin{array}{c} \lambda_{1}\\ .\\ \lambda_{i}\\ .\\ \lambda_{n} \end{array}\right)={\left(\begin{array}{cccc} C_{1} & x_{1c} & y_{1c} & A_{1}\\ . & . & . & .\\ C_{i} & x_{ic} & y_{ic} & A_{i}\\ . & . & . & .\\ C_{n} & x_{nc} & y_{nc} & A_{n} \end{array}\right)}_{\left(n\times 8\right)}$$

where n denotes the number of parts, $C_{i}$ is the category of part i, $x_{ic}\;\mathrm{and}\;y_{ic}$ are the center coordinates of part i, and $A_{i}$ is the relative size of the 2D BBox pixel area between parts.

The size of *M* is n×8 instead of n×4 because we used one-hot encoding to represent the type of part i ($C_{i}$). One-hot encoding slightly improves the accuracy of our framework and will be described in Section 5. To facilitate the design of the yaw angle decoding network, the size of the information matrix must be fixed. Due to the self-occlusion of the vehicle [47,48], the maximum number of parts that can be observed at one time is about six, so we designed the following method to fix the information matrix at a size of 6×8. For n≤6, 6−n empty part codes are added, $\lambda_{null}=\left[0,0,0,0,0,0,0,0\right]$, at the end. For *n* > 6, n−6 part codes are randomly discarded. Eventually, the six part codes are shuffled (which may contain empty part codes) to make the network more adaptive. In this way, the information matrix of the specified size is obtained through the part-encoding network.

### 4.2. Yaw Angle Decoding Network

The yaw angle decoding network is used to decode the information matrix to obtain the vehicle pose. Our input information matrix is obtained by refining the information in the image. It has high information purity, so the strategy adopted here extracts richer information by enhancing the network width rather than extracting deeper semantic information by increasing the network depth.

#### 4.2.1. Design of the Network Structure

**Figure 9:**
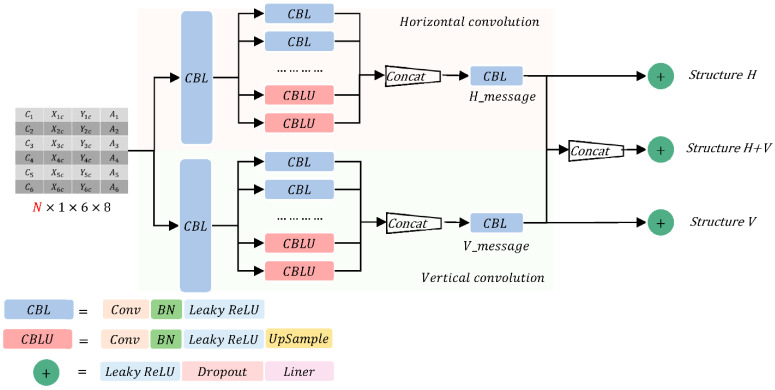
Structure of the pose-decoding network. The convolutional layer uses asymmetric convolution, and the combination of information is extracted by convolutional kernels in two directions. After testing, among the three network structures, structure H achieves the best network performance with the same number of parameters; therefore, we adopted structure H as the network structure.

We used a convolutional neural network to extract the information of yaw angles embedded in the information matrix (Figure 9). Given the significant difference between the information represented by rows and columns in the matrix, we used an asymmetric convolutional kernel [49] in horizontal and vertical convolutions. The horizontal convolution extracts the information composed of different combinations of parts. We used a 1×8 convolution kernel to compress the information matrix into a 6×1 matrix with c channels. The six elements in the 6×1 matrix represent the complete information of six parts. Next, the information between different parts is upsampled [50] and fused into the information H_message from the combination of different parts. Moreover, the vertical convolution extracts the information from different information types (category, position, and size) of all the parts. We used a 6×1 convolution kernel to compress the information matrix into a 1×8 matrix with c channels. The eight elements in the 1×8 matrix represent the types of information of all parts. Next, all parts with different types are sampled and fused into the information V_message from the combination of different types. In Section 5, we will describe the design of a controlled trial to demonstrate the use of the H_message and V_message. Experimental results show that the network with H_message alone outperforms that with V_message alone and that which combines H_message and V_message.

#### 4.2.2. Design of Loss Functions

In addition to the design of the network structure, to obtain accurate estimation results, we needed an appropriate loss function design to make the network converge in a given direction. The design of the loss function is challenging due to the periodicity of the angle [51]. Hence, we designed a total of three loss functions for testing.

(a) SSE Loss Function of Angle:

The SSE (sum of squared errors) loss function of angle uses the SSE between the predicted angle and the labeled angle as the loss:

(5) $$L=\sum_{j\in N}||Ang_{j}^{pre}-Ang_{j}^{{}_{label}}|{|}^{2}$$

where N denotes a set of instrumental data, and $Ang_{j}^{pre}$ and $Ang_{j}^{label}$ are the predicted yaw angle and label yaw angle for data j, respectively. However, because the yaw angles fall in the range of 0 to 360, the angles are continuous rather than abrupt in real-world conditions. Yaw angles of 0° and 360° indicate the same physical meaning. However, yaw angles of 0° and 360° are the two results with the greatest difference. To solve the above problem, we designed the SSE loss function of the minimum angle.

**Figure 10:**
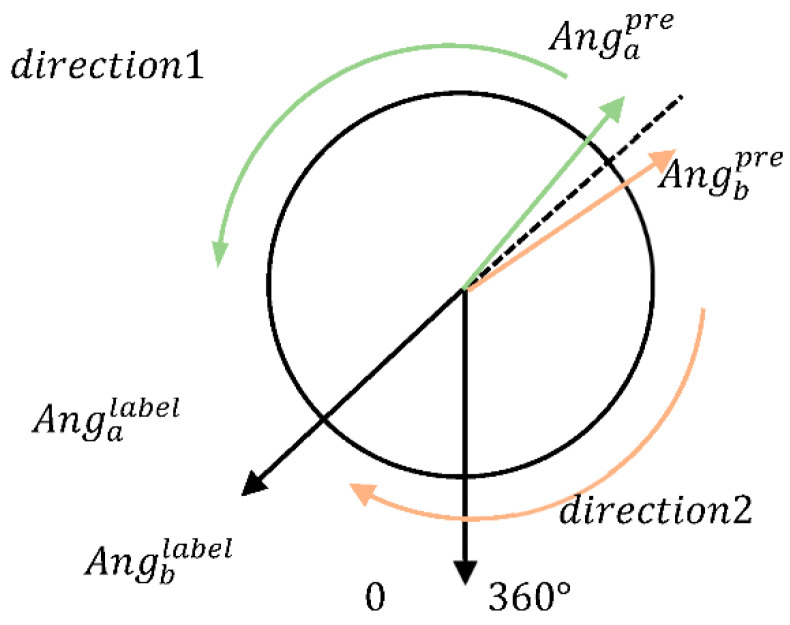
Illustration of the convergence direction of Equation (6).

(b) SSE Loss Function of Minimum Angle:

The SSE loss function of the minimum angle uses the SSE between the predicted angle and the label angle in the real world as the loss:

(6) $$\begin{array}{c} L=\sum_{j\in N}||f(Ang_{j}^{pre},Ang_{j}^{{}_{label}})|{|}^{2}\\ f(a,b)=\{\begin{array}{cc} \left|a-b\right| & \left|a-b\right|\leq 180\;or\left|a-b\right|>360\\ 360-\left|a-b\right| & 180<\left|a-b\right|\leq 360 \end{array} \end{array}$$

where f(a,b) is a function of the real-world angular deviation of a and b. The deviation calculated by f(a,b) is closer to real-world conditions and can be used as a loss to improve the model accuracy. However, the training results show that such a loss function complicates model convergence because the parameter gradient decreases randomly during network training. Let a and b be two similar pieces of data with consistent labels (such data are common in datasets). During the training, their predicted results ($res_{j}=\left|Ang_{j}^{pre}-Ang_{j}^{label}\right|$) are likely to approximate $res_{a}\langle 180,res_{b}\rangle 180$. The gradient descent will change the model in the direction of loss reduction, but a network with the same input will converge in opposite directions (Figure 10). This creates a problem in the network, with the network parameters in oscillation, making model convergence difficult.

**Figure 11:**
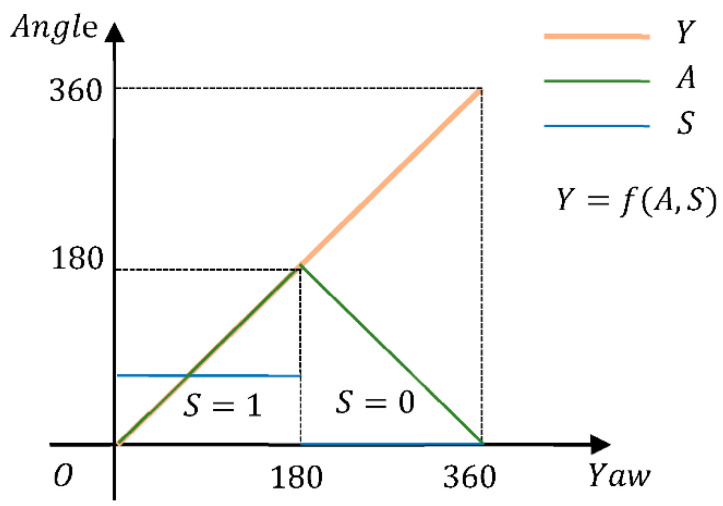
Illustration of the function between *Y*, *A*, and *S*.

(c) SSE Loss Function of Adding the Sign:

To solve the above problem, we proposed an SSE loss function of adding the sign. We split yaw angle Y from 0 to 360° into two parameters, S and A (Equation (7)); the function of Y,A,S is shown in Figure 11. The loss function (L) is composed of$\;L_{A}$ and $L_{S}$ (Equation (8)). In this way, the mutations of Y at 0 and 360° can be transformed into the mutations of *S* at 0 and 1. As shown in Figure 11, unlike the yaw angle parameter (*Y*), parameter A is continuous. The prediction of S by a deep neural network is relatively simple; it only needs to classify the vehicle left-facing pose (0≤Y<180) as S=1 and the vehicle right-facing pose (180≤Y<360) as S=0. The accuracy of our model is dramatically improved by representing the angle in this way:

(7) $$\left(A,S\right)=\{\begin{array}{cc} \left(Y,1\right) & Y<180\\ \left(360-Y,0\right) & Y\geq 180 \end{array}$$

(8) $$L=L_{A}+L_{S}=\sum_{j\in N}\left(||A_{j}^{pre}-A_{j}^{{}_{label}}|{|}^{2}\right)+\sum_{j\in N}\left(||S_{j}^{pre}-S_{j}^{{}_{label}}|{|}^{2}\right)$$

where S is the sign position indicating the range of Y, and $L_{A}\;\mathrm{and}\;L_{S}$ represent the angle loss and sign loss, respectively.

## 5. Experiment and Analysis

Experiments were designed to validate our model presented in Section 4, including experiments on the part-encoding network and the yaw angle decoding network. The part-encoding network and the yaw angle decoding network are independent of each other when training the model. The input to the part encoding network is the images of various vehicles and the bounding box annotations of the vehicles and vehicle parts. The inputs to the yaw angle decoding network are the information matrices, which are obtained by encoding the manual labels of the vehicle parts, and the yaw angle, which is collected by the GPS. Because any type of advanced object detector is acceptable for the part-encoding network, in this section, we do not focus on its optimal design but on designing the network structure and the network parameters of the yaw angle decoding network. We compared the yaw angle decoding network structures and verified the methods proposed here by designing an ablation experiment.

### 5.1. Evaluation Metrics

To assess the effectiveness of the model, appropriate evaluation metrics are required. For the part-encoding network, we used the classic mean average precision (mAP) metric [52], which characterizes the accuracy and precision of the object detection model. For the yaw angle decoding network, we used the average value of the yaw angle deviation, denoted as E, to characterize the model accuracy. To evaluate the correct rate of angle estimation, we proposed the passing rate of yaw angle deviation ($EP_{a}$), which indicates that the percentage of angle prediction error is less than a° in the prediction. In a driving scenario, the variation of yaw angle is greater than 10° whether the vehicle is changing lanes or steering. We consider that the prediction error of yaw angle estimation is tolerable within 10°, so we adopted $EP_{10}$ as the primary evaluation metric. We also tested the metric $EP_{5}$ to test the model correctness with subtle angle changes.

### 5.2. Experiment with Object-Encoding Network

With the object-encoding network, the information of the parts from the images is extracted to form the information matrix. The Yaw Angle Dataset including 17,258 data point was used to train the vehicle and vehicle part object detectors. The training results are shown in Table 2. The mAP metric of the result achieves an accuracy of 99.6%, satisfying our coding requirements.

### 5.3. Experiment with Yaw Angle Decoding Network

We use the Yaw Angle Dataset (which contains 15,863 data point) to train the yaw angle decoding network proposed in Section 4.2. With the aim of obtaining the vehicle yaw angle directly from the image, we used the yaw angle and image datasets in the Yaw Angle Dataset to evaluate the model performance. The Val Yaw Angle Dataset was used to evaluate the performance of the yaw angle decoding network, which is composed of 10% selected data from the Yaw Angle Dataset that were not involved in model training, with 1586 information matrices. The image dataset consisting of 1915 vehicle images was used to evaluate the performance of YAEN. In these images, we considered both realistic driving scenes and the variation of yaw angle, including scenes representing following, meeting, and figure-eight loop scenarios for both vehicles.

#### 5.3.1. Ablation of Network Structure

Many attempts were made to design the structure of the yaw angle decoding network, and the performance results of different models were obtained by training different models many times. The main structures include the horizontal convolution structure (H), the vertical convolution structure (V), and the fusion structure of horizontal and vertical convolution (H + V). The horizontal convolution kernel with a larger horizontal size is used to extract the complete information of each component. The vertical convolution kernel with a larger vertical size is used to extract information consisting of combinations of different parts. We maintained the sizes of all model training parameters below 3 M(million) and trained all network structures using the Adam optimizer [53]. Moreover, the learning rates and training epochs of different models were kept consistent. The performance of different models on the two datasets are shown in Table 3.

According to Table 3, horizontal convolution outperforms vertical convolution in extracting the vehicle yaw angle. model3, which is obtained by fusing the vertical extracted information into the horizontal convolution model, has achieves a lower model performance compared with model2, which includes horizontal convolution. This result indicates that the combination of information from different parts extracted by horizontal convolution conveys more accurate information of yaw angle than the combination of different types of information from all parts extracted by vertical convolution. It is for this reason that method H performs better than method H + V with roughly the same amount of parameters.

#### 5.3.2. Ablation of Tricks

To improve the model performance, we also identified several useful method in addition to network structure adjustment. To verify the effectiveness of these methods, we conducted an ablation experiment. The main methods are one-hot encoding and varying loss functions. One-hot encoding adopts the one-hot code to indicate the type of each part. For example, there are five part types: none, wheel, headlight, taillight, and rearview mirror. When one-hot encoding is not used, the expressions of the wheel and taillight are 1 and 3, respectively; when one-hot encoding is adopted, the expressions become [0,1,0,0,0] and [0,0,0,1,0], respectively. We expect one-hot encoding to better represent the type of parts and the design of different loss functions to solve the convergence problem of the model, as specified in Section 4.2.2. Similarly, we controlled the sizes of all model training parameters below 3 M (million) and fixed the network structures as horizontal convolution (H). All network structures were trained with an Adam optimizer [53], and the learning rates and training epochs of different models were kept consistent. The performance results of the ablation experiment are shown in Table 4.

Comparing model2, model4, and model5 in Table 4, we found that the problems of abrupt angle and model convergence can be effectively solved by the loss function using the SSE of adding the sign. Compared with the loss function using angle SSE, the accuracy (E and $EP_{10}$) of the loss function using the SSE of adding the sign are improved by 3.68° and 5.17%, respectively. On the contrary, the loss function using the SSE of minimum angle correctly represents the deviation between the predicted angle and the actual angle in the real world, which makes model convergence difficult and obtains the worst result. Results from model5 and model6 show that the use of one-hot encoding only slightly improves the network performance, more effectively conveying the information of part types to the network and improving the model accuracy (E) by 0.24° and $EP_{10}$ by 0.31%. The best model is model6, with an average angle estimation error of 3.09° and an $EP_{10}$ value up to 96.45% on the Image dataset.

Benefiting from our network design, our model can achieve a fast detection speed without requiring excessive computational resources. The YAEN model requires minimal video memory (less than 2 GB) and is very fast using the available graphics card, RTX2070s. We performed two tests of YAEN. The first test evaluated the detection speed of the yaw angle decoding network, the input of which is the information matrix. The other test examined the detection speed of the whole model, the input of which is an image with a resolution of 1200×1920×3. The test results of the two tests are shown in Table 5. Clearly, the yaw angle decoding network in YAEN consumes few resources and can achieve a detection speed of 97FPS in the whole model test, satisfying real-time requirements.

### 5.4. Visualization of Detection

**Figure 12:**
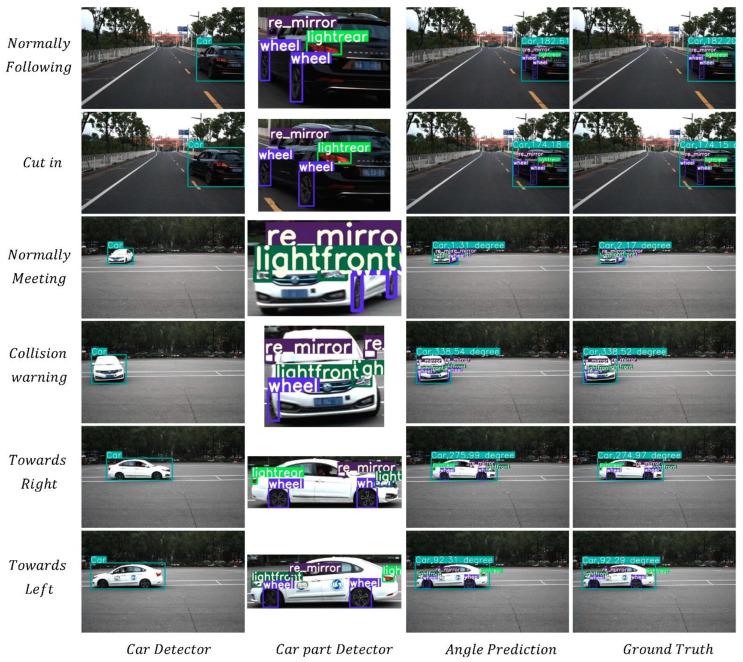
Evaluation of vehicle yaw angle estimation in different scenes. The first to the fourth columns show the detection results of the vehicle, the vehicle parts, the estimated value of yaw angle, and the label value of yaw angle, respectively.

We selected typical driving scenes to visualize the detection, including a same-direction following scene and an opposite-direction meeting scene (Figure 12). In the same-direction following scene, the relative yaw angle deviation between the observed vehicle in a normal state and the observing vehicle is about 180° (first row in Figure 12). When the observed vehicle aims to cut into the main lane from other lanes, its yaw angle relative to the observing vehicle changes (second row of Figure 12). The change in yaw angle can be detected before the vehicle leaves its lane, enabling prediction of the movements of surrounding vehicles in advance, which is essential to improving driving safety. In the opposite-direction meeting scene, the movements of the surrounding vehicles can be predicted by accurately predicting the yaw angle. In addition to the typical road scenes, we also estimated the vehicle yaw angle in scenarios not common in the normal driving process to reflect the performance of yaw angle detection (last two lines in Figure 12). Our model can accurately predict the yaw angles of all kinds of attitudes.

**Figure 13:**
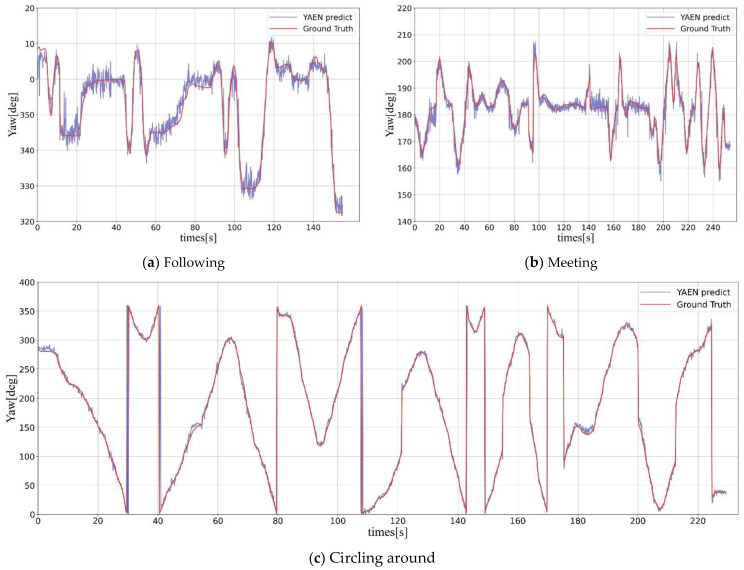
Line graph of YAEN-estimated yaw angle and label yaw angle in typical scenes. Curves of the labeled and predicted values of the vehicle yaw angle in (**a**) a following scene, $E=1.67,\;EP_{5}=95.97\%,\;EP_{10}=99.66\%$; (**b**) a meeting scene, $E=1.82,\;EP_{5}=95.87\%,\;EP_{10}=99.56\%$; and (**c**) a figure-eight loop scene,$\;E=3.40,\;EP_{5}=75.54\%,\;EP_{10}=96.73\%.$

The estimation of yaw angle by YAEN is not only accurate but also very stable. We evaluated the stability of YAEN in several scenes, including real roads and closed driving fields (Figure 13). Figure 13 shows the line graphs of the actual yaw angle and the YAEN-predicted yaw angle in several scenes. Results show that YAEN can accurately and stably predict vehicle yaw angle. In particular, the correct estimation of estimation error less than 10° is more than 99%. This accurate and stable estimation can support high-level decision tasks. For example, we can judge whether the vehicle is out of control by observing the change in yaw angle and predict the future vehicle trajectory according to the yaw angle. This is important information for driverless vehicles.

### 5.5. Analysis and Discussion

We did not evaluate the performance of our algorithm on a publicly available dataset due to deficiencies in the accuracy of the current pose estimation dataset; however, in the future, we will make our dataset publicly available. To further illustrate the superiority of our method, we performed the following analysis.

The proposed framework based on part arrangement for accurate yaw angle estimation is sensitive to the vehicle structure but not to the appearance of the vehicle (e.g., color, texture, etc.) because the information on vehicle parts is obtained by abstraction of the part-encoding network, which does not contain color and texture information about vehicle parts. Thanks to the improvement of object detection performance in recent years, the part-encoding network is able to meet our requirement, as shown in Table 2. We selected two of the most common vehicles encountered in daily life: SUVs and sedans. Because of the use of our asymmetric convolution and the design of the loss function with adding the sign, our model performs well. The accuracy of our model is shown in Table 4. The average predicted yaw error for both models is below 3.1 degrees. The robustness of our model is shown in Figure 13; the predicted and true values are in a relatively stable state for following and meeting scenarios. Thus, our proposed framework for prediction of the vehicle attitude based on part arrangement is able to predict the vehicle’s yaw angle accurately and stably.

## 6. Conclusions and Future Work

In this paper, we found that object parts are strongly represented in the object pose and proposed a framework for estimation of vehicle yaw angle based on the part arrangement, which was proven to be effective by our dataset. The proposed framework is divided into two steps: a part-encoding network and a yaw angle decoding network. With the part encoder network, we refined the features of the vehicle parts in the image to obtain an information matrix. This information matrix contains only the type, center point pixel coordinates, and size information of the vehicle parts but not the edge and texture information of the parts. This operation that makes the design of our yaw angle decoding network lightweight and the model rapid. According to experimental testing, YAEN can achieve a detection speed of 97FPS on the available graphics card, RTX2070s. In the yaw angle decoding network, we proposed a network structure using non-pairwise convolution and a loss function with adding the sign for the angle regression problem, resulting in an accuracy improvement of more than 5%. According to network design and loss function design our model achieves satisfactory performance. To verify the performance of our algorithm, we constructed the Yaw Angle Dataset, and the experimental results show that YAEN can achieve an accuracy of 96.45% for prediction errors below 10° in real driving scenario. In conclusion, YAEN can detect the yaw angle of a vehicle quickly and accurately.

Despite YAEN’s satisfactory performance, it can still be improved. Currently, YAEN mainly considers single-vehicle yaw angle detection, whereas yaw angle detection of multiple vehicles is a challenging task for YAEN. Due to the possibility of mutual occlusion between multiple vehicles, YAEN cannot always divide each vehicle part correctly, which leads to the loss of yaw angle accuracy of multiple vehicles. As a result of limitations with respect to the experimental site, most of the data we collected are horizontal roads, so detection accuracy was not tested on ramps. In future work, we will consider using virtual datasets to synthesize data for testing in scenarios such as ramps.

## Figures and Tables

**Table 1:** The function of the equipment and performance parameters.

Device	Quantity	Mounting Location	Function	Performance Parameters
OXTS GPS RT3000 v2	1	Observing vehicle	Provide data on the latitude, longitude, yaw angle, roll angle, pitch angle, and speed of the observing vehicle	Location: 0.01 m Angle: 0.03°
OXTS GPS RT3000 v2	1	Observed vehicle	Provide data on the latitude, longitude, yaw angle, roll angle, pitch angle, and speed of the observed vehicle	Location: 0.01 m Angle: 0.03°
OXTS RT-BASE	1	Near the test site	Improve the positioning accuracy of OXTS GPS RT3000 v2	-
FLIR GS3-U3	2	Observing vehicle	Provide pictures of the observed vehicle from the perspective of the observing vehicle	Resolution: 1920 × 1200 × 3 FPS: 160
Velodyne VLP-32C	1	Observing vehicle	Provide the point cloud of the observed vehicle from the perspective of the observing vehicle	Horizontal angular resolution: 0.1°to 0.4°
TL-AP450GP	1	Near the test site	Construct a local area network for communication between devices	2.4 GHz, 450 Mbps

**Table 2:** Object detector network training results. Note: average accuracy of all classes: 0.996; l_f: headlights, l_r: taillights, and r_m: rearview mirrors.

Method	Car	Wheel	l_f	l_r	r_m	mAP
Object Detector	0.996	0.995	0.996	0.996	0.995	0.996

**Table 3:** The performance of different network structures on the Val yaw angle dataset and Image dataset. Note: E: average error of estimated angle; $EP_{a}\;$(%): percentage of prediction error of angles less than a° in the prediction; V: vertical convolution structure; H: horizontal convolution structure; H + V: the fusion structure of V and H.

Model	Method	Size	Val Yaw Angle Dataset			Image Dataset		
			E(°)	$EP_{5}\left(\%\right)$	$EP_{10}\left(\%\right)$	E(°)	$EP_{5}\left(\%\right)$	$EP_{10}\left(\%\right)$
model1	V	2.57 M	10.41	58.01	79.00	10.06	59.74	81.46
model2	H	2.82 M	**6.76**	**68.35**	**88.59**	**7.01**	69.77	**90.97**
model3	H + V	2.94 M	7.30	66.02	86.76	7.87	**70.08**	90.29

**Table 4:** The performance of different methods on the Val yaw angle dataset and image dataset. Note: L_1: loss function using SSE loss, L_2: loss function using the SSE loss of minimum angle, L_3: loss function using the SSE loss of adding the sign; OH: use of one-hot encoding to represent the part type.

Model	Method	Tricks	Size	Val Yaw Angle Dataset			Image Dataset		
				E(°)	$EP_{5}\left(\%\right)$	$EP_{10}\left(\%\right)$	E(°)	$EP_{5}\left(\%\right)$	$EP_{10}\left(\%\right)$
model2	H	L_1	2.82 M	6.76	68.35	88.59	7.01	69.77	90.97
model4	H	L_2	2.82 M	6.69	54.29	76.17	6.41	55.35	80.05
model5	H	L_3	2.82 M	3.64	78.31	**93.63**	3.33	82.56	96.14
model6	H	L_3+OH	2.82 M	**3.40**	**78.69**	**93.63**	**3.09**	**83.19**	**96.45**

**Table 5:** Evaluation results of the YAEN model detecting speed on the RTX2070s computing platform.

Platform	FPS: Yaw Angle Decoder			FPS: Complete Model		
	Fastest	Average	Slowest	Fastest	Average	Slowest
RTX2070s	503	497	465	98	97	93

## Data Availability

Not applicable.
